# The role of advanced nuclear reactors and fuel cycles in a future energy system

**DOI:** 10.1093/pnasnexus/pgae030

**Published:** 2024-01-23

**Authors:** Kasia Kornecki, Catherine F Wise

**Affiliations:** Board on Energy and Environmental Systems, National Academies of Sciences, Engineering, and Medicine, 500 5th St. NW, Washington DC 20001, USA; Board on Energy and Environmental Systems, National Academies of Sciences, Engineering, and Medicine, 500 5th St. NW, Washington DC 20001, USA

## Abstract

Nuclear power has been an important part of the US electricity system since the 1950s and continues to be a major source of low-carbon electricity today. Despite having low emissions, high grid reliability, and an excellent track record of safety, nuclear power also demands significant time and upfront capital to deploy, can struggle to compete economically with other generation sources, has intrinsic proliferation risk by relying on fissile material for fuel, and generates radioactive waste for which there are currently no disposal sites. Given the emissions and energy security benefits of having nuclear as part of the energy mix, advanced nuclear technologies have garnered significant interest and investment in recent years. Advanced reactor designs differ from the current operating fleet and have several potential advantages, including lower cost, faster construction, smaller size, inherent safety features, and lower waste yields. Yet, many challenges related to their deployment remain, and overcoming them will dictate whether or not new nuclear technologies become a material element of the future energy infrastructure. This article synthesizes the opportunities and barriers to deploying advanced nuclear reactors and their associated fuel cycles as described in two National Academies consensus reports. It highlights the consensus recommendations that could allow these new technologies to reach commercial success as part of a long-term decarbonization strategy.

## Introduction

Nuclear power has supplied electricity to the US grid since the 1950s and currently accounts for 18.2% of total US electricity generation (95.5 GWe), and about 50% of low-carbon electricity. Most analyses of electricity system decarbonization in the United States show that the current fleet of nuclear plants needs to remain online for several decades if climate goals are to be met, despite many of them having waning license lifetimes and some facing economic challenges from competition with lower-cost alternatives. Further, the lack of a spent fuel disposal facility creates a problem for the once-through fuel cycle (OTC)^1^ used in the United States. Confronting the contradictory ideas on nuclear power—that it could have environmental benefits as an essential element of meeting climate and emissions goals, but also environmental and safety hazards due to the lack of a permanent solution for nuclear waste—creates a fraught policy and investment landscape for this energy technology. Although safety has long been a source of concern after the Chernobyl and Fukushima accidents, the inability to expand the nuclear reactor fleet has fundamentally been due to financial, regulatory, and construction issues. Nonetheless, deploying new and advanced nuclear reactors^2^ to address concerns about climate change and energy security has garnered considerable interest in recent years. While the pathway for advanced nuclear is far from clear, its future is a foundational question for deep decarbonization and the evolution of the electricity system in the United States.

To better understand the proposed benefits of advanced reactors, as well as the technical, societal, economic, and regulatory outlook for these reactors and their associated fuel cycles, the National Academies of Sciences, Engineering, and Medicine carried out two parallel consensus studies. One of the studies, *Laying the Foundation for New and Advanced Nuclear Reactors in the United States*, analyzed the opportunities and barriers for new nuclear technologies to play a role in a future net-zero-emissions energy system.^3^ The other study, *Merits and Viability of Different Nuclear Fuel Cycles and Technology Options and the Waste Aspects of Advanced Reactors*, evaluated nuclear fuel cycle and waste management options for advanced reactors, as well as their nonproliferation implications and security risks.^4^ The two studies shared one committee member and several National Academies staff members, but otherwise operated independently and produced independent findings and recommendations that are the consensus of each individual committee.

Here, we present a synthesis of these two studies. Our objective is to provide a comprehensive outlook on the many issues associated with making advanced nuclear a material element of a future decarbonized energy system. Over the short term, electricity system decarbonization efforts will primarily focus on increased deployment of wind, solar, and natural gas, and retirement of coal. The medium-to-long term will require additional resources that can provide on-demand and/or constant electricity and energy. This article relies on the findings and recommendations of two consensus committees that identified the critical hurtles that nuclear must overcome, both today and in the future. It begins by providing context about the role of nuclear power in the current energy system and identifying variables that will impact its future viability. These variables then serve as the basis of discussion on developing a practicable fuel cycle; deploying a meaningful number of new reactors; ensuring adequate nonproliferation, security, and safeguards measures; and deploying reactors abroad.

## Nuclear power's role in the current and future energy system

In the coming decades, demand for electricity—particularly low-carbon electricity—is projected to increase as decarbonization efforts spur electrification of transportation, industry, and buildings. Advanced nuclear reactors could meet some of this new demand; however, projected timelines for deployment of other clean energy technologies suggest a longer-term role for advanced nuclear. The first demonstrations of advanced reactors expect to come online in the 2030s, and subsequent deployment of reactors to support increased electrification and electricity demand in the 2040s and 2050s will depend on market competitiveness. But many variables will impact the viability of advanced nuclear technologies in future energy markets, including: reactor technology research, reactor and fuel cycle safety, fuel availability and disposition, project management and construction, workforce, alternative energy applications, federal funding, financing and credibility, community engagement and acceptance, security and safeguards, nonproliferation considerations, and international markets.

## Advanced reactor designs

Advanced nuclear reactors span a wide range of designs, including small modular light water reactors (LWRs) and nonwater-cooled designs, such as sodium- and lead-cooled fast reactors, gas- and fluoride salt-cooled high-temperature reactors (FHRs), gas-cooled fast reactors (GFRs), and fluoride- and chloride-based molten-salt-fueled and cooled reactors. Proposed reactor designs employ a variety of coolants, fuel types and forms, and neutron spectra, and are at different levels of technology readiness, as depicted in Table 1. There are, however, several notable similarities across many of the proposed reactor designs: requirement for high-assay low-enriched uranium (HALEU) fuel, smaller size than large LWRs (e.g. 100 s vs. 1,000 MWe), more inherent and passive safety features than current operating reactors, and initial use of a OTC. Implications of these design choices are discussed in the following sections.

**Table 1.** Design features and technology readiness of new and advanced nuclear reactors under development.

Reactor design	Coolant	Fuel type	Enrichment	Neutron spectrum	Initial fuel cycle	Technology experience	Technology gaps	Example(s)
Small modular LWR	Light water	UO_2_	LEU	Thermal	Once through	Evolution from currently operating LWRs	Develop and qualify unique plant components	NPM (NuScale)
Liquid metal fast reactor	Liquid sodium or lead	U metal alloy, UN	HALEU	Fast	Once through	Several small SFRs operating worldwide	Qualify annular metal fuel and advanced steel alloys Perform source term experiments to reduce conservatisms	Natrium (TerraPower-GEH), ARC-100 (ARC), SEALER-55 (LeadCold), LFR (Westinghouse)
High-temperature gas reactor	Helium	UCO TRISO	HALEU	Thermal	Once through	Several small HGTRs operating worldwide	Qualify fuel and graphite (<1,100 K outlet temp designs) Qualify materials used in heat exchanger and other components (>1,100 K outlet temp designs)	Xe-100 (X-Energy), SC-HGTR (Framatome)
Fluoride salt-cooled reactor	F-based salt	UCO TRISO	HALEU	Thermal	Once through	FHR designed, reduced-scale prototype planned for demonstration	Demonstrate corrosion/control for FLiBe-based salt in presence of neutron field Demonstrate materials for strength, corrosion resistance, and irradiation stability in operation Demonstrate tritium migration and radioactivity control Demonstrate passive safety systems	KP-X FHR (Kairos)
Heat-pipe-cooled reactor	Heat pipe	U metal, UC TRISO	HALEU	Thermal or fast	Closed	ORNL experiments operated without power conversion systems	Demonstrate corrosion control for salts, tritium migration and control, materials for long-term operation Demonstrate passive safety systems	eVinci (Westinghouse), Aurora (Oklo)
Molten-salt-fueled-cooled reactor	F- or Cl-based salt	U or Th fluoride, U or Pu chloride	LEU or HALEU	Thermal or fast	Once-through or closed	LANL space reactor demonstrated concept at reduced power scale	Develop compact PCU operation and integration with heat-pipe core cooling Develop autonomous control and instrumentation Demonstrate passive safety systems	IMSR-400 (Terrestrial Energy), SSR-W (Moltex), LFTR (Flibe Energy)
Gas-cooled fast reactor	Helium	UC	HALEU	Fast	Once through	No reactor ever built	Qualify fuel, clad, and structural materials for safety, radiation damage Demonstrate passive safety systems	EM^2^ (GA)

Overall technology readiness level (TRL) is in order from highest to lowest going from top to bottom of the table. UO_2_, uranium dioxide; UCO, uranium oxycarbide; TRISO , TRistructural ISOtropic; LEU, low-enriched uranium; HALEU, high-assay low-enriched uranium; LWR, light water reactor; SFR, sodium-cooled fast reactor; HGTR, high-temperature gas reactor; FHR, fluoride salt-cooled high-temperature reactor; ORNL, Oak Ridge National Laboratory; LANL, Los Alamos National Laboratory.

The overall technology readiness of a reactor design depends on a number of factors, including previous experience with the design and actions required to address remaining technology gaps. Technology gaps for more mature designs—small modular LWRs, small sodium-cooled fast reactors (SFRs), high-temperature gas reactors (HTGRs)—deal with regulatory qualification of new systems and performance regimes. Technology gaps for less mature designs—GFRs, molten salt reactors (MSRs), large SFRs, and FHRs—involve demonstrating viability and performance of key reactor features. Thus, while more mature designs may be technically ready for demonstration by 2030, less mature concepts will likely not be ready for demonstration until after 2030 (Finding 2-5(1)). Of the advanced reactor designs, small modular LWRs are furthest along toward connecting to the grid, as they leverage existing reactor and fuel cycle infrastructure and have received government support for over a decade (Finding 5(2)).

## The front end of the fuel cycle

As illustrated in Table 1 above, advanced reactor developers propose to use a wide variety of fuel types (e.g. ^235^U, ^239^Pu, ^233^U(Th)) and fuel forms [e.g. U metal, metal alloy, U nitride, U carbide, TRistructural ISOtropic (TRISO) particles, liquid fuel salts]. The United States has no commercial fuel fabrication capability for HALEU-based or nonuranium dioxide fuels, though US-based X-energy is constructing a facility to fabricate HALEU-based TRISO particles in Oak Ridge, TN, which is expected to be operational by 2025 and will initially produce 8 MT of TRISO fuel per year (3, 4). Establishing a domestic HALEU supply chain to meet the needs of advanced reactors is particularly important, as the only current commercial supplier of HALEU is Russia, which poses national and energy security implications. In line with Recommendation C in NASEM (2), DOE has entered into a cost-sharing agreement with Centrus to construct enrichment centrifuges for HALEU and has committed to downblending high-enriched uranium at the Savannah River Site to produce HALEU.

Both NASEM reports recognize the critical importance of fuels and materials testing to support advanced reactor development and improve both technical and cost performance. A coordinated program involving the DOE, US Nuclear Regulatory Commission (NRC), Electric Power Research Institute (EPRI), nuclear industry, national laboratories, and universities should target aggressive research and development (R&D) goals for improving fuels and materials performance (Recommendation 2-2(1)). Additionally, Congress and DOE should provide or ensure access to materials testing and fuels qualification capabilities required by advanced reactor developers (Recommendation B(2)).

## Developing and deploying advanced nuclear reactors

In addition to incorporating new fuels and materials, advanced reactor developers are proposing new deployment scenarios to meet the needs of an evolving energy system. Examples include deploying modular plants to match power capacity requirements of different customers; repurposing retiring fossil generation sites to benefit from existing transmission infrastructure; providing nonelectricity services such as thermal energy storage, high-temperature process heat, and low-temperate heat for district heating or desalination; and using microreactors at remote sites. While electricity production is expected to remain the predominant use-case for nuclear power, generation of thermal energy for industrial applications—especially hydrogen production—could be an important new opportunity. More R&D on system integration, operations, safety, community acceptance, regulatory risks, and market size for industrial applications of nuclear is needed, and DOE should partner with industry support groups like EPRI and reactor vendors to analyze these opportunities (Recommendation 5-1(1)).

### Reactor and fuel cycle safety

For any deployment scenario, reactor design, or fuel cycle choice, safety is a key consideration. As mentioned above, advanced reactor designs incorporate more passive and inherent safety features than current large LWRs. The combinations of fuel, coolant, and moderator in non-LWRs can provide inherent favorable safety characteristics, such as physical stability, high heat capacity, and negative reactivity feedback. Both LWR and non-LWR designs incorporate engineered passive safety systems that require no AC emergency power and fewer operator actions. Together, these design attributes could make it simpler, more reliable, and more cost effective to fulfill safety functions, while also being more tolerant of human error (Finding 2-1(1)). Reactor designers will need to collect test data at appropriate scales and operating experience to demonstrate these key safety functions, which requires new testing and demonstration facilities (Finding 2-2(1)). While advanced reactor developers have focused on improving the safety of the reactors themselves, less attention has been paid to advanced fuel cycles, all of which—and particularly those that reprocess and recycle spent fuel—will introduce additional safety and environmental considerations compared to the OTC with LWRs (Finding 9(2)).

### Economic competitiveness

A major challenge for developing and deploying advanced reactors is economics. Advanced reactors need to be cost-competitive with other low-carbon electricity sources, provide energy applications beyond electricity, and/or present another strong value proposition that yields investments (Finding 4-1(1)). Electricity system modeling suggests that $2,000–$4,000/kW costs would make advanced nuclear competitive regardless of other market conditions, while costs of $4,000–$6,000/kW could be competitive in the case of higher power system costs or high demand for nonelectricity products (Finding 3-1(1)). While industry has the primary responsibility for commercializing advanced reactors, government support will be required for completing demonstrations and developing finance structures and market incentives. Programs such as the Advanced Reactor Demonstration Program (ARDP) should be maintained, but to ensure efficient use of demonstration funds, DOE should develop milestone-based decision-making criteria that guide program and budget decisions and allow for deliberate down-selection of technologies (Recommendation 4-3(1); Recommendation A (2)). Additionally, federal and state governments should provide appropriately tailored financial incentives for industry to use as part of a commercialization plan, with tools that may vary by state, locality, or market type (Recommendation 4-4(1)). Credibility also remains a key investment barrier—in order to mobilize investors in equity and debt markets, all aspects of project risk need to be minimized.

Recent nuclear power plant construction in the United States has suffered from significant cost and schedule overruns, primarily related to onsite civil work, which can represent 40–50% of the overnight capital costs of large LWRs. Consequently, some advanced reactor developers are seeking to employ a “product-based” approach to construction, where reactor units are manufactured in a factory and then delivered to a site, with a goal of improving learning, cost and schedule performance, quality, and speed and scale of deployment (Finding 6-11(1)). Nonetheless, given the importance of onsite civil work for any approach to reactor deployment, DOE should increase its R&D efforts in advanced construction technologies for nuclear and make these technologies available to recipients of ARDP awards and decrease the costs associated with construction (Recommendation 6-8(1)). Developing the relevant skills and workforce will also be critical, and nuclear owners/operators should “consider establishing a consortium or joint venture to pursue construction on behalf of the group, thereby enabling the creation and maintenance of the necessary skilled technical engineering personnel” (Recommendation 6-2(1)). Additionally, DOE should establish a whole-of-government initiative that would, in collaboration with labor organizations, industry, regulatory agencies, and other support agencies, identify gaps in critical skills and fund training programs to address them (Recommendation 6-1(1)).

### Regulatory updates for advanced reactor deployment

There are two licensing pathways for new commercial reactors in the United States: Part 50, a two-stage licensing process, and Part 52, a one-stage process that combines the construction and operating license application. Both of these pathways are based on a foundation of technology-specific prescriptive requirements that can be enhanced or relaxed based on probabilistic risk analysis. Since both of these pathways are technology specific and based on the LWR design, new prescriptive requirements would need to be developed for each non-LWR technology under these existing pathways—a considerable challenge that would require enhanced capacity within the NRC. Given this challenge, there is interest in developing an all-technology-inclusive licensing pathway, Part 53, which the Nuclear Energy Innovation and Modernization Act directed the NRC to develop by 2027. Although applicants can currently pursue license applications under Parts 50 or 52 with exemptions for technology differences, without clarity on what new regulatory guidelines will entail, advanced nuclear reactors cannot play a role in the future energy system. Ambiguity in the regulatory outlook for non-LWR technologies affects the ability of investors and other stakeholders to plan projects. The NRC needs to promptly establish a clear definition of regulatory requirements for new reactors based on their differences from existing LWRs (Recommendation 7-1(1)). As one example, as of 2023 August 15, the NRC has indicated that they will provide for a scalable approach to determining the size of the emergency planning zone, which could lead to the consideration of smaller safety zones for advanced reactors in some circumstances. Additionally, Congress should increase funding to the NRC on the order of tens of millions of dollars to facilitate this work, so as to not rely solely on the fee base recovered from the applicant (Recommendation 7-1(1)).

### Societal acceptance of nuclear power

Societal acceptance of nuclear power will also significantly impact the potential widespread deployment of advanced reactors. The nuclear industry needs to engage with communities being considered for reactor deployment using the following best practices: “(i) a participatory process of site selection; (ii) the right for communities to veto or opt out (within agreed-upon limits); (iii) some form of compensation granted for affected communities; (iv) partial funding for affected communities to conduct independent technical analyses; (v) efforts to develop a partnership to pursue the project between the implementer and local community; and (vi) an overriding commitment to honesty” (Recommendation 8-5(1)). Implementing these practices does not guarantee success but does increase its likelihood, and the nuclear industry should account for the additional time and financial resources for community engagement in its planning (Recommendation 8-5(1)). Final decisions about hosting new nuclear projects will always ultimately be made at the community level.

## The back end of the fuel cycle

**Fig. 1.**
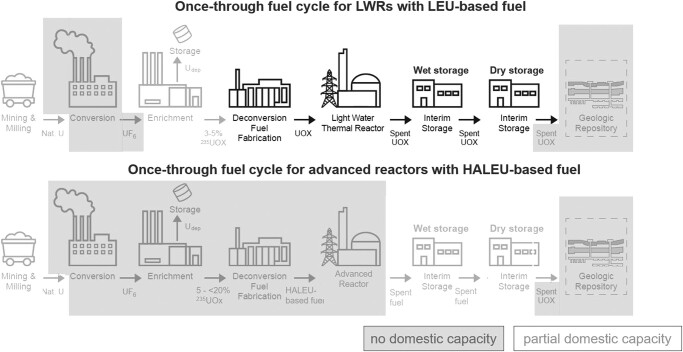
Components of the OTC for LWRs using LEU fuel (top) and for advanced reactors using HALEU-based fuel (bottom). Those components for which there is no current domestic capacity are indicated in gray boxes, while those for which there is partial domestic capacity are indicated by light gray (2).

Management of spent nuclear fuel on the back end of the fuel cycle includes interim storage, waste transportation, any reprocessing or recycling operations, and final geologic disposal. Different fuel cycle options manage spent fuel in different ways: the OTC involves direct disposal of spent fuel, monorecycle involves reprocessing to separate plutonium and uranium for reuse once in a reactor, and multirecycle involves reprocessing to separate plutonium and minor actinides for continuous recycling in a reactor. There are many variations on this basis set of three fuel cycle options depending on which isotopes are separated from spent fuel, recycled in the reactor, and sent to permanent disposal. As indicated in Table 1, most advanced reactor developers are opting initially for a OTC, which aligns with the recommendation that “the current US policy of using a OTC with the direct disposal of commercial spent nuclear fuel into a repository should continue for the foreseeable future” (Recommendation D(2)).^5^ Figure 1 depicts current domestic capabilities for a OTC, showing both the existing system with low-enriched uranium (LEU) fuel and LWRs and the proposed future system involving HALEU-based fuel and advanced reactors.

Even with the continuation of an OTC, advanced reactors introduce new considerations and requirements to the back end of the fuel cycle because, compared to uranium oxide spent fuel from LWRs, their fuel wastes differ in amount (mass or volume), chemical composition, radionuclide inventory, thermal power, and durability in a disposal environment (Finding 13)(2). As such, there exists little experience or technical ability for managing many of the waste streams from proposed advanced reactors at a commercial scale (Finding 15)(2). For example, HTGRs would produce much larger volumes of spent fuel, SFRs would generate irradiated sodium waste and sodium-bonded spent fuel, MSRs would produce radioactive off-gases and spent fuel salt waste, and many reactor designs would contribute large quantities of irradiated graphite from moderators or reflectors. Advanced reactor developers are not focusing on waste management and disposal, largely because there is no incentive for them to do so under current US policy (Finding 12(2)).

A geologic repository for the disposal of highly radioactive waste will be required regardless of fuel cycle choice, yet the United States has no clear path forward for siting, licensing, and constructing one, following years of delays in planning and progress. To address this issue, Congress should establish a single-mission entity responsible for managing and disposing nuclear wastes (Recommendation G(2)). This entity should handle cradle-to-grave management and disposal of nuclear wastes with continuity of leadership and funding; be funded by users of nuclear-generated electricity, with funds collected in an escrow account not subject to annual appropriations; and immediately initiate the process of site selection for a geologic repository using appropriate technical criteria and acceptable public engagement methods (Recommendation G(2)). It should also support R&D on the behavior of spent fuel from advanced reactors in various geologic environments, recycling and reuse options for irradiated graphite, and management and disposal of unique waste streams from advanced reactors (Recommendation H(2)). Concurrently, NRC, DOE, and EPA should develop regulations and standards for a generic repository and geologic disposal of the new types of spent fuel and waste forms that would be produced from advanced reactors (Recommendation I(2)). While advanced reactors could conceptually reduce the inventory of transuranic elements in existing spent fuel, the significant resources and time required make this option impractical (Finding 14(2)); in fact, the introduction of advanced reactors would “do little, if anything, to eliminate the need to manage and dispose of nuclear waste” ((2), p. 157). Therefore, the “immediate-future focus of the US nuclear waste management and disposal program should be planning for the geologic disposal of the existing spent fuel” (Recommendation J(2)).

While there are no anticipated “showstopper” issues for transporting and storing waste streams from advanced reactors and fuel cycles, more R&D is required to characterize their performance envelopes and develop stable waste forms compatible with NRC regulations (Finding 17(2)). Integrating HALEU (both fresh and irradiated) into the fuel cycle will require (i) criticality experiments for >5% enrichment to support benchmarking analyses, (ii) assessments of the feasibility of using type 30B containers for transport, and (iii) criticality, thermal, and shielding assessments for storage and transportation (Finding 18(2)). DOE should collaborate with the nuclear industry and advanced reactor developers, through avenues such as EPRI's Extended Storage Collaboration Program, to identify and address packaging issues for storing and transporting waste streams from advanced reactors (Recommendation K(2)).

## Nonproliferation, security, and safeguards considerations for advanced reactors

The different design features and fuel cycles proposed for advanced reactors introduce unique nonproliferation considerations and safeguards and security risks that need to be managed. Specific issues requiring attention include: use of special nuclear materials such as HALEU, plutonium, minor actinides, and thorium and uranium-233; use of fuels with different material concentrations and chemical and physical forms as compared to LWR oxide fuel (e.g. pebble-bed technologies and molten salt fuel); new refueling cycles, including online refueling and long-lived once-through cores; and implementation of fuel cycles involving reprocessing and reuse of separated nuclear materials. Because HALEU will be used in most advanced reactor designs, NASEM (2) recommended that:

- The National Nuclear Security Administration coordinate with DOE-NE to assess proliferation and security risks associated with expanded global use of HALEU, in parallel with an international effort fostered by the US government and facilitated by the International Atomic Energy Agency (IAEA), to examine and address these risks (Recommendation M).
- The NRC initiate a rulemaking to address security and material accounting measures for HALEU and other attractive materials (Recommendation O).

New deployment scenarios, such as remote operation, reduced staffing, and transportable facilities, also pose potential security risks. Greater use of automation, sensors, and controls could increase cybersecurity risks. Advanced reactor developers will need clear guidance on security requirements in order to accommodate them in reactor designs and staffing and operations plans. Congress should provide the NRC with additional funding to evaluate security guidelines and, if needed, expedite rulemaking on modified security requirements (Recommendation 9-1(1)).

To facilitate development and application of effective safeguards, the US government should authorize IAEA access through the eligible facilities list to advanced reactor systems for which it does not currently have safeguards experience (Recommendation N(2)). Additionally, the IAEA and DOE should “identify funding, personnel, regulatory analyses, and technology gaps for pilot programs in international safeguards for advanced reactors” and “develop cost incentive-based programs to encourage early-adopter vendor participation in safeguards development” (Recommendation 9-3(1)).

## International nuclear deployment

Although initially planning for domestic deployments, many US-based advanced reactor vendors intend to deploy their designs internationally, which will require Nuclear Cooperation Agreements with additional countries and may involve modifications to nuclear export controls. To compete in an international market, US vendors will need more options for financing the export and deployment of their reactors, which could include mechanisms such as Build Own Operate and Build Own Operate Transfer (Finding 10-4(1)). The vendors will need an effective and competitive financing package consisting of federal grants, loans, and loan guarantees, as well as private equity and debt financing, and the Executive Branch should work with the private sector to develop such a package (Recommendation 10-2(1)).

Along with financial support, support for research in domestic and international safeguards and security, international engagement, and licensing assessment is also important for international deployment of advanced reactors. The United States should develop a plan for capacity building in partner countries, which “should include partnering with US reactor vendors to develop a safety, safeguards, and security “package,” where the United States and the vendor could offer customized support to a host country for developing and implementing new safety, safeguards, and security arrangements” (Recommendation 9-5(1)).

## Conclusions

Economy-wide decarbonization to mitigate climate change presents a significant opportunity for advanced nuclear reactors, but many challenges must be overcome for them to contribute to the future low-carbon energy system. Addressing these challenges will require sustained effort and robust financial support by the US Congress, federal agencies, nuclear industry, and financial community. A vast array of advanced reactor and fuel cycle concepts have been proposed, and no single advanced technology can concurrently provide all of the potential benefits of advanced reactors. The US government and industry will have to decide which attributes of advanced reactors and fuel cycles best align with US energy needs without increasing proliferation risks, having an adverse impact on the environment, or imposing an unacceptable economic burden on current and future generations. No matter what decisions are made about advanced reactor and fuel cycle development, the United States should immediately begin planning for geologic disposal of spent fuel and high-level waste from existing reactors, with a first step being to establish a single-mission entity responsible for the management and disposal of nuclear waste.

The energy system is in a state of transition, and significant changes to energy generation and demand are expected by 2030. Nuclear and other low-carbon technologies will be vying for a place in the market over the coming decades. While there is uncertainty in what the future electricity system will look like, advanced nuclear has the potential to serve a variety of longer-term opportunities, both on the grid and in other applications. “The race against climate change is both a marathon and a sprint” (1)—although advanced nuclear reactors are sprinting toward establishing a commercial foundation this decade, deployments in the late 2030s, 2040s, 2050s, and beyond will make up the marathon.

## Data Availability

No original data were used in the writing of this manuscript. This article synthesizes two consensus study reports that are available for free at the urls below: NASEM (National Academies of Sciences, Engineering, and Medicine) (2). *Merits and Viability of Different Nuclear Fuel Cycles and Technology Options and the Waste Aspects of Advanced Nuclear Reactors*. Washington, DC: The National Academies Press. https://doi.org/10.17226/26500 NASEM (1). *Laying the Foundation for New and Advanced Nuclear Reactors in the United States*. Washington, DC: The National Academies Press. https://doi.org/10.17226/26630

## Footnotes

1 The OTC involves the single use of nuclear fuel, followed by direct disposal of the spent fuel.

2 Advanced reactors include all designs that differ from traditional GW-scale light water reactors. This includes small modular light water reactors and designs utilizing different moderators and coolants. As defined in the Nuclear Energy Innovation Capabilities Act of 2017 (NEICA), advanced reactors could provide a number of benefits compared to the existing reactor fleet, namely “(i) inherent safety features, (ii) lower waste yields, (iii) greater fuel utilization, (iv) superior reliability, (v) resistance to proliferation, (vi) increased thermal efficiency, and (vii) the ability to integrate into electric and nonelectric applications” (P.L. 115–248).

3 This study originated from interest within the National Academies and in Congress, and it was funded by a donation to the National Academy of Engineering from Dr James Truchard, with additional funding from DOE’s Office of Nuclear Energy (DOE-NE). The study began in December 2020 and released its report in April 2023. The study committee and consensus report authors were Richard A. Meserve (Chair), Ahmed Abdulla, Todd Allen, Jaquelin Cochran, Michael L. Corradini, Richard Cupitt, Leslie Dewan, Heather Feldman (until April 2022), Michael Ford, Kirsty Gogan, Ning Kang, Allison M. Macfarlane, David K. Owens, James Rispoli, Rachel Slaybaugh (until May 2022), Sola Talabi, and Steven J. Zinkle.

4 This study responded to Congressional mandates in the Further Consolidated Appropriations Act of 2020 (P.L. 116–194) and Consolidated Appropriations Act of 2021 (P.L. 116–126) and was funded by DOE-NE. The study began in September 2020 and released its report in November 2022. The study committee and consensus report authors were Janice Dunn Lee (Chair), Patricia A. Baisden (Vice Chair), Rodney C. Ewing (Vice Chair, until 2022 July), Margaret S. Chu, Paul T. Dickman, Craig S. Hansen, John C. Lee (until 2022 September), Edwin S. Lyman, Allison M. Macfarlane, Albert J. Machiels, Christophe Poinssot, Jeffery D. Semancik, Ken B. Sorenson, Jasmina Vujic (until 2022 September), Nathalie A. Wall, and Houston G. Wood (until 2021 September).

5 The recommendation also notes that DOE should continue R&D on recycling and transmutation so as not to preclude such options further in the future.
